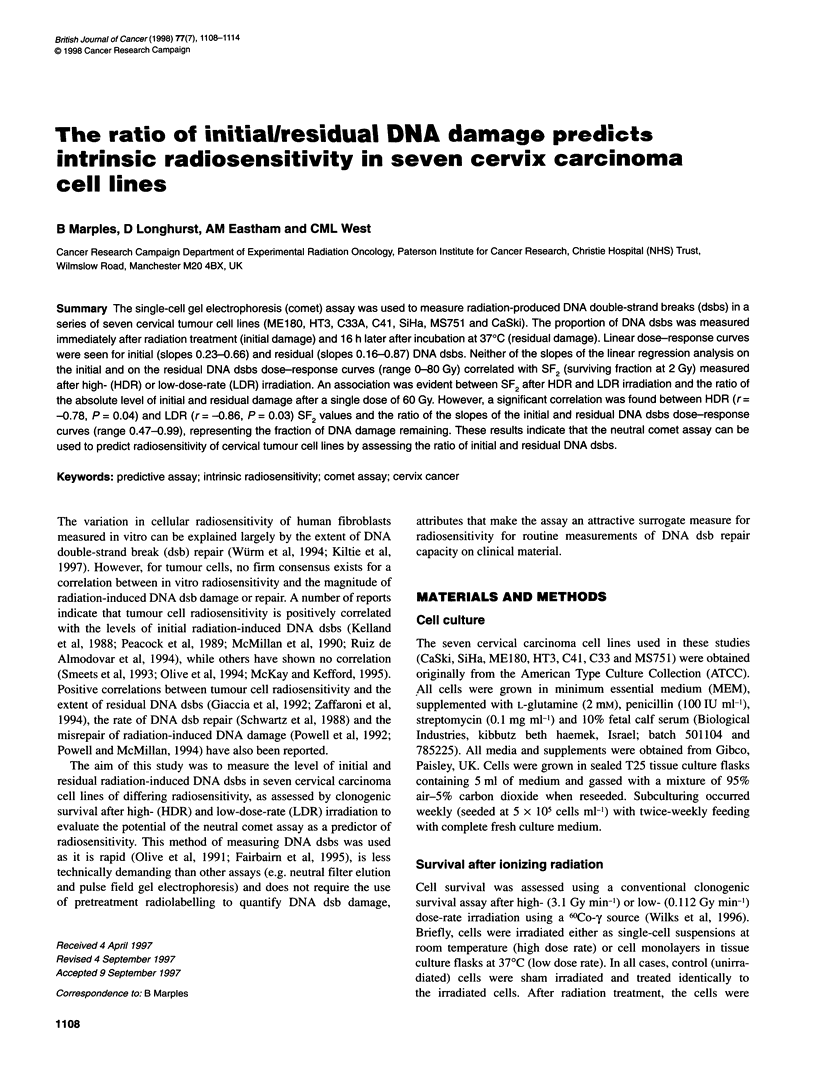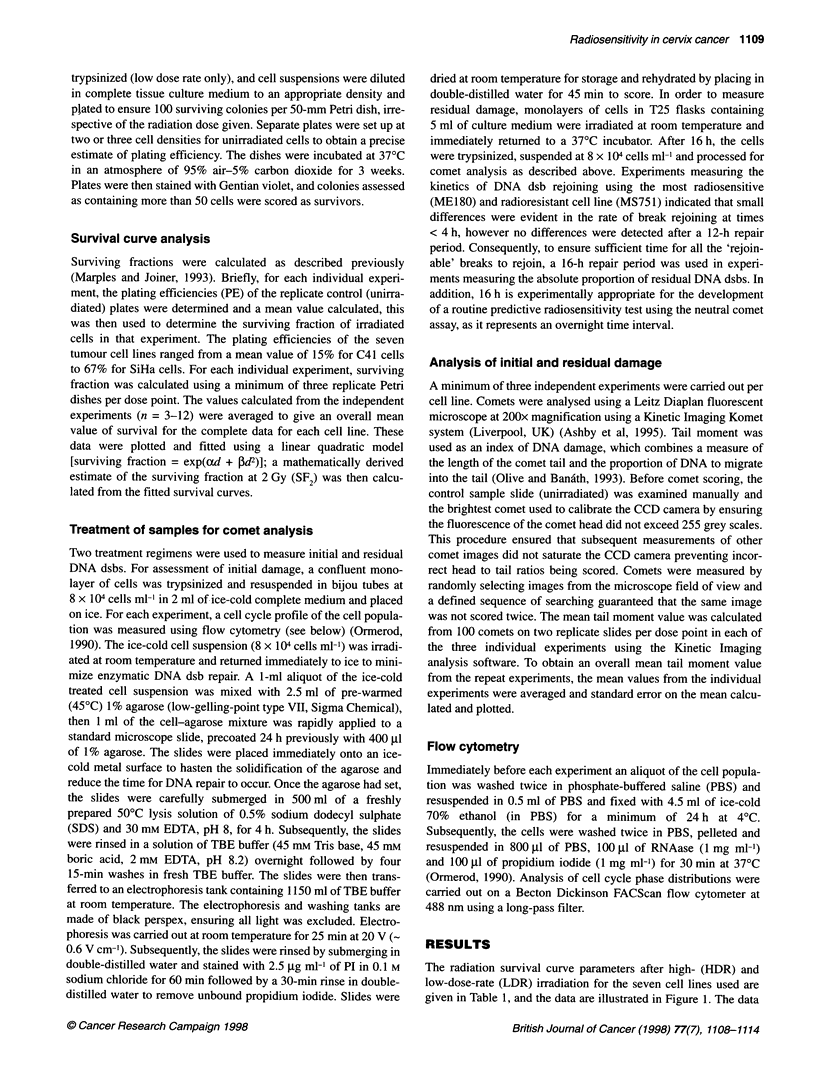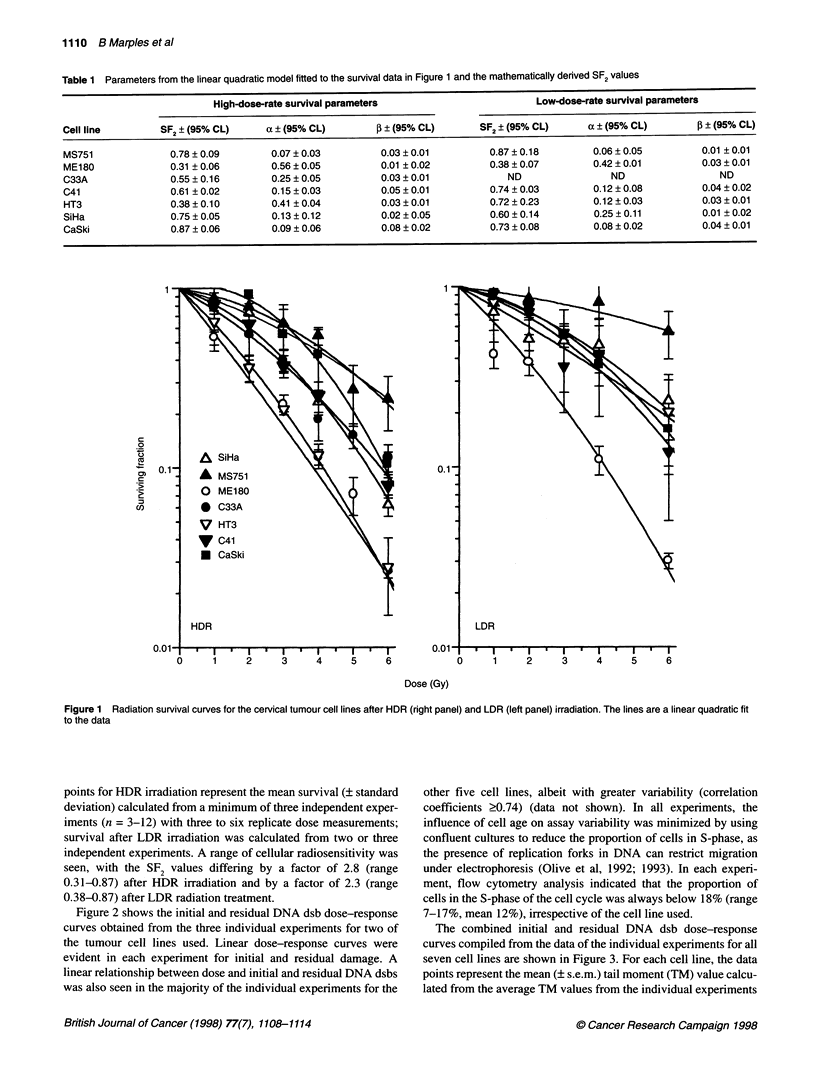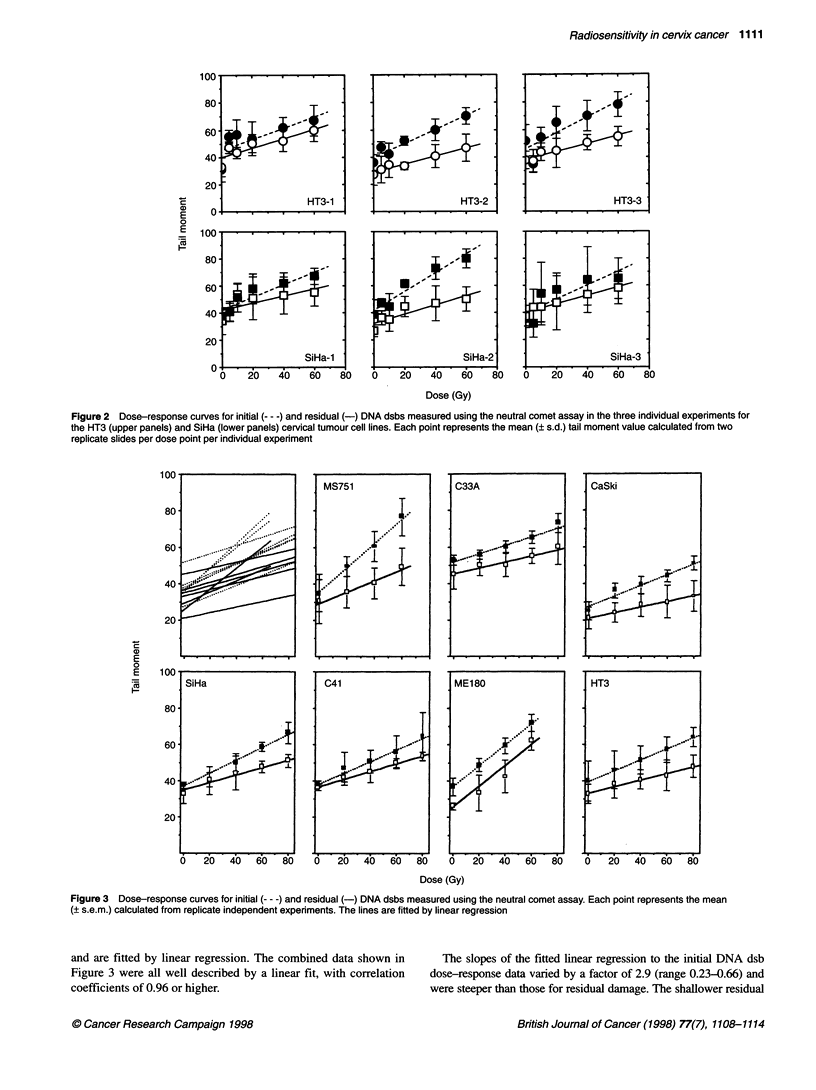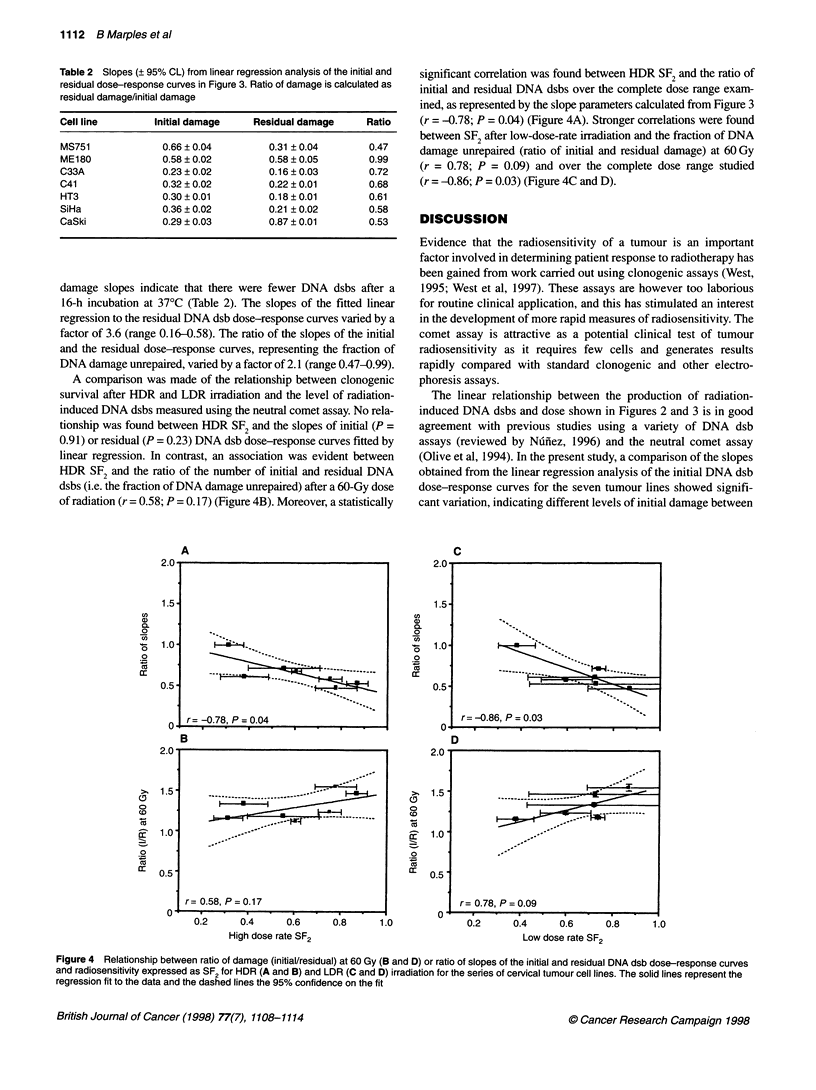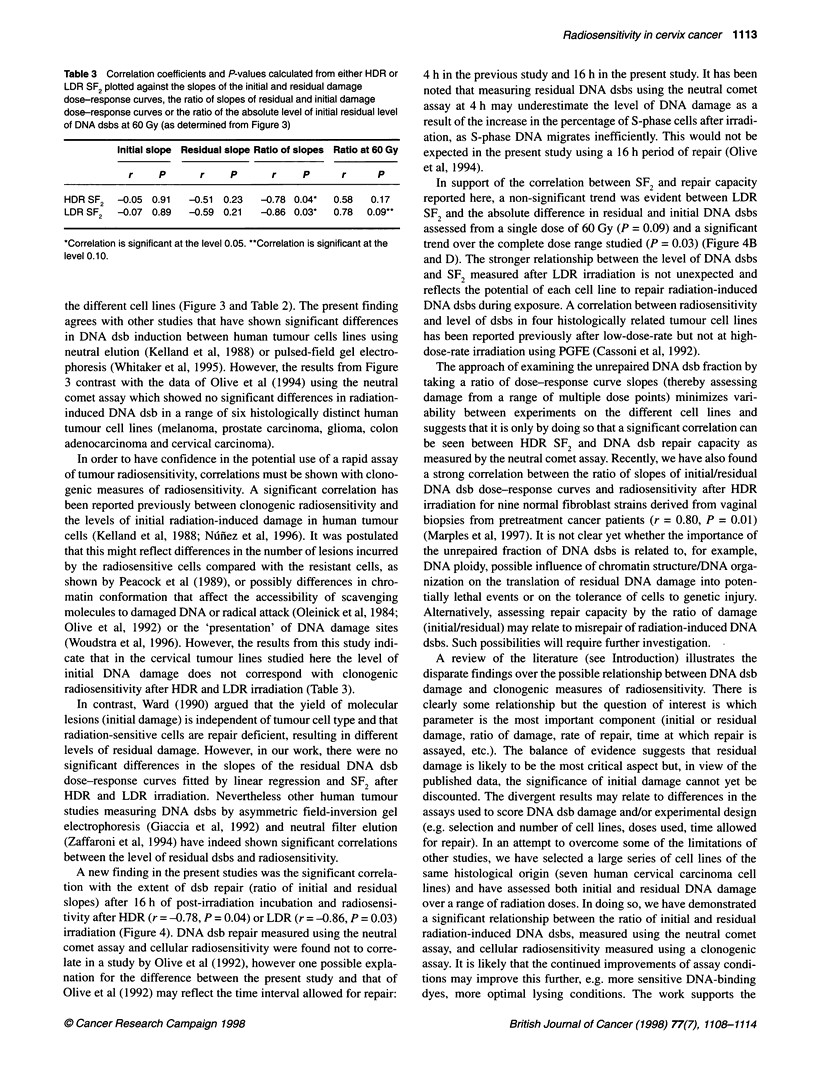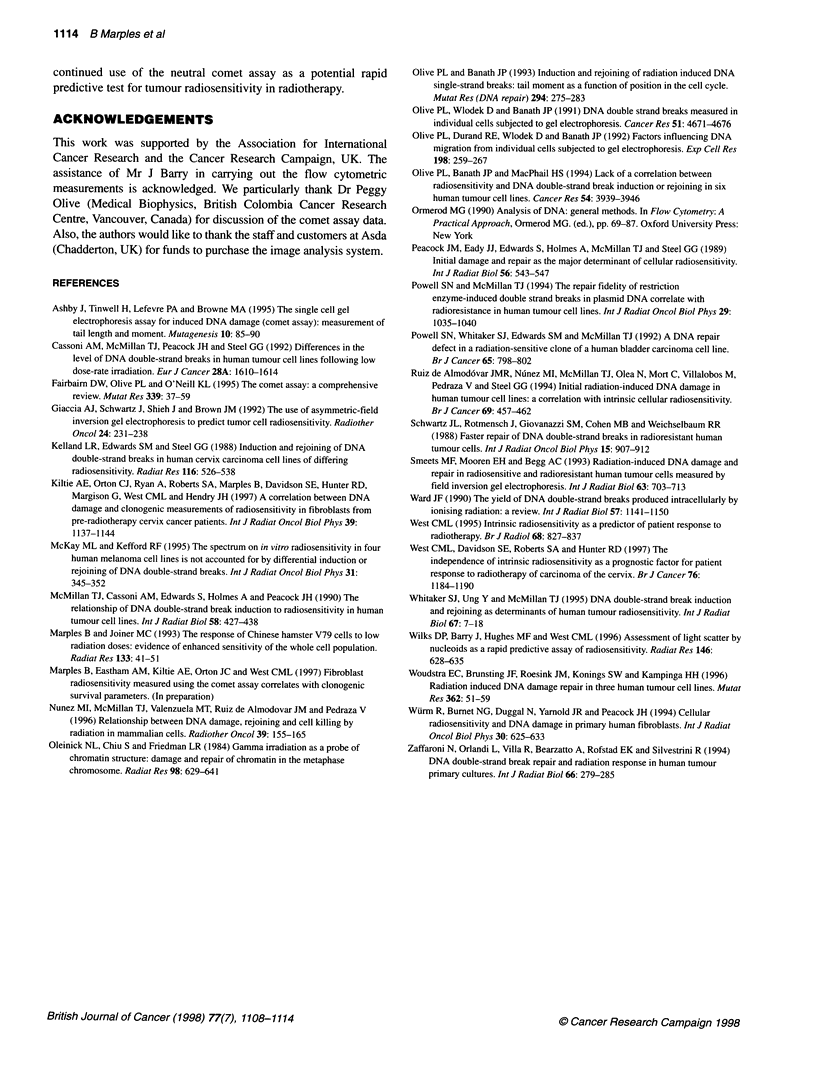# The ratio of initial/residual DNA damage predicts intrinsic radiosensitivity in seven cervix carcinoma cell lines.

**DOI:** 10.1038/bjc.1998.184

**Published:** 1998-04

**Authors:** B. Marples, D. Longhurst, A. M. Eastham, C. M. West

**Affiliations:** Cancer Research Campaign Department of Experimental Radiation Oncology, Paterson Institute for Cancer Research, Christie Hospital (NHS) Trust, Manchester, UK.

## Abstract

The single-cell gel electrophoresis (comet) assay was used to measure radiation-produced DNA double-strand breaks (dsbs) in a series of seven cervical tumour cell lines (ME180, HT3, C33A, C41, SiHa, MS751 and CaSki). The proportion of DNA dsbs was measured immediately after radiation treatment (initial damage) and 16 h later after incubation at 37 degrees C (residual damage). Linear dose-response curves were seen for initial (slopes 0.23-0.66) and residual (slopes 0.16-0.87) DNA dsbs. Neither of the slopes of the linear regression analysis on the initial and on the residual DNA dsbs dose-response curves (range 0-80 Gy) correlated with SF2 (surviving fraction at 2 Gy) measured after high- (HDR) or low-dose-rate (LDR) irradiation. An association was evident between SF2 after HDR and LDR irradiation and the ratio of the absolute level of initial and residual damage after a single dose of 60 Gy. However, a significant correlation was found between HDR (r= -0.78, P = 0.04) and LDR (r = -0.86, P = 0.03) SF2 values and the ratio of the slopes of the initial and residual DNA dsbs dose-response curves (range 0.47-0.99), representing the fraction of DNA damage remaining. These results indicate that the neutral comet assay can be used to predict radiosensitivity of cervical tumour cell lines by assessing the ratio of initial and residual DNA dsbs.


British Journal of Cancer (1998) 77(7), 1108–1114
© 1998 Cancer Research Campaign

### The ratio of initial/residual DNA damage predicts intrinsic radiosensitivity in seven cervix carcinoma cell lines

**B Marples, D Longhurst, AM Eastham and CML West**

Cancer Research Campaign Department of Experimental Radiation Oncology, Paterson Institute for Cancer Research, Christie Hospital (NHS) Trust, Wilmslow Road, Manchester M20 4BX, UK

**Summary** The single-cell gel electrophoresis (comet) assay was used to measure radiation-produced DNA double-strand breaks (dsbs) in a series of seven cervical tumour cell lines (ME180, HT3, C33A, C41, SiHa, MS751 and CaSki). The proportion of DNA dsbs was measured immediately after radiation treatment (initial damage) and 16 h later after incubation at 37°C (residual damage). Linear dose–response curves were seen for initial (slopes 0.23–0.66) and residual (slopes 0.16–0.87) DNA dsbs. Neither of the slopes of the linear regression analysis on the initial and on the residual DNA dsbs dose–response curves (range 0–80 Gy) correlated with $SF_2$ (surviving fraction at 2 Gy) measured after high- (HDR) or low-dose-rate (LDR) irradiation. An association was evident between $SF_2$ after HDR and LDR irradiation and the ratio of the absolute level of initial and residual damage after a single dose of 60 Gy. However, a significant correlation was found between HDR ($r = -0.78$, $P = 0.04$) and LDR ($r = -0.86$, $P = 0.03$) $SF_2$ values and the ratio of the slopes of the initial and residual DNA dsbs dose–response curves (range 0.47–0.99), representing the fraction of DNA damage remaining. These results indicate that the neutral comet assay can be used to predict radiosensitivity of cervical tumour cell lines by assessing the ratio of initial and residual DNA dsbs.

**Keywords:** predictive assay; intrinsic radiosensitivity; comet assay; cervix cancer

The variation in cellular radiosensitivity of human fibroblasts measured in vitro can be explained largely by the extent of DNA double-strand break (dsb) repair (Würm et al, 1994; Kiltie et al, 1997). However, for tumour cells, no firm consensus exists for a correlation between in vitro radiosensitivity and the magnitude of radiation-induced DNA dsb damage or repair. A number of reports indicate that tumour cell radiosensitivity is positively correlated with the levels of initial radiation-induced DNA dsbs (Kelland et al, 1988; Peacock et al, 1989; McMillan et al, 1990; Ruiz de Almodovar et al, 1994), while others have shown no correlation (Smeets et al, 1993; Olive et al, 1994; McKay and Kefford, 1995). Positive correlations between tumour cell radiosensitivity and the extent of residual DNA dsbs (Giaccia et al, 1992; Zaffaroni et al, 1994), the rate of DNA dsb repair (Schwartz et al, 1988) and the misrepair of radiation-induced DNA damage (Powell et al, 1992; Powell and McMillan, 1994) have also been reported.

The aim of this study was to measure the level of initial and residual radiation-induced DNA dsbs in seven cervical carcinoma cell lines of differing radiosensitivity, as assessed by clonogenic survival after high- (HDR) and low-dose-rate (LDR) irradiation to evaluate the potential of the neutral comet assay as a predictor of radiosensitivity. This method of measuring DNA dsbs was used as it is rapid (Olive et al, 1991; Fairbairn et al, 1995), is less technically demanding than other assays (e.g. neutral filter elution and pulse field gel electrophoresis) and does not require the use of pretreatment radiolabelling to quantify DNA dsb damage, attributes that make the assay an attractive surrogate measure for radiosensitivity for routine measurements of DNA dsb repair capacity on clinical material.

*Received 4 April 1997*
*Revised 4 September 1997*
*Accepted 9 September 1997*

*Correspondence to:* B Marples

### MATERIALS AND METHODS

### Cell culture

The seven cervical carcinoma cell lines used in these studies (CaSki, SiHa, ME180, HT3, C41, C33 and MS751) were obtained originally from the American Type Culture Collection (ATCC). All cells were grown in minimum essential medium (MEM), supplemented with L-glutamine (2 mM), penicillin (100 IU $ml^{-1}$), streptomycin (0.1 mg $ml^{-1}$) and 10% fetal calf serum (Biological Industries, kibbutz beth haemek, Israel; batch 501104 and 785225). All media and supplements were obtained from Gibco, Paisley, UK. Cells were grown in sealed T25 tissue culture flasks containing 5 ml of medium and gassed with a mixture of 95% air–5% carbon dioxide when reseeded. Subculturing occurred weekly (seeded at $5 \times 10^5$ cells $ml^{-1}$) with twice-weekly feeding with complete fresh culture medium.

### Survival after ionizing radiation

Cell survival was assessed using a conventional clonogenic survival assay after high- (3.1 Gy $min^{-1}$) or low- (0.112 Gy $min^{-1}$) dose-rate irradiation using a $^{60}$Co-γ source (Wilks et al, 1996). Briefly, cells were irradiated either as single-cell suspensions at room temperature (high dose rate) or cell monolayers in tissue culture flasks at 37°C (low dose rate). In all cases, control (unirradiated) cells were sham irradiated and treated identically to the irradiated cells. After radiation treatment, the cells were trypsinized (low dose rate only), and cell suspensions were diluted in complete tissue culture medium to an appropriate density and plated to ensure 100 surviving colonies per 50-mm Petri dish, irrespective of the radiation dose given. Separate plates were set up at two or three cell densities for unirradiated cells to obtain a precise estimate of plating efficiency. The dishes were incubated at 37°C in an atmosphere of 95% air–5% carbon dioxide for 3 weeks. Plates were then stained with Gentian violet, and colonies assessed as containing more than 50 cells were scored as survivors.

### Survival curve analysis

Surviving fractions were calculated as described previously (Marples and Joiner, 1993). Briefly, for each individual experiment, the plating efficiencies (PE) of the replicate control (unirradiated) plates were determined and a mean value calculated, this was then used to determine the surviving fraction of irradiated cells in that experiment. The plating efficiencies of the seven tumour cell lines ranged from a mean value of 15% for C41 cells to 67% for SiHa cells. For each individual experiment, surviving fraction was calculated using a minimum of three replicate Petri dishes per dose point. The values calculated from the independent experiments ($n$ = 3–12) were averaged to give an overall mean value of survival for the complete data for each cell line. These data were plotted and fitted using a linear quadratic model [surviving fraction = $\exp(\alpha d + \beta d^2)$]; a mathematically derived estimate of the surviving fraction at 2 Gy ($SF_2$) was then calculated from the fitted survival curves.

### Treatment of samples for comet analysis

Two treatment regimens were used to measure initial and residual DNA dsbs. For assessment of initial damage, a confluent monolayer of cells was trypsinized and resuspended in bijou tubes at $8 \times 10^4$ cells $ml^{-1}$ in 2 ml of ice-cold complete medium and placed on ice. For each experiment, a cell cycle profile of the cell population was measured using flow cytometry (see below) (Ormerod, 1990). The ice-cold cell suspension ($8 \times 10^4$ cells $ml^{-1}$) was irradiated at room temperature and returned immediately to ice to minimize enzymatic DNA dsb repair. A 1-ml aliquot of the ice-cold treated cell suspension was mixed with 2.5 ml of pre-warmed (45°C) 1% agarose (low-gelling-point type VII, Sigma Chemical), then 1 ml of the cell–agarose mixture was rapidly applied to a standard microscope slide, precoated 24 h previously with 400 µl of 1% agarose. The slides were placed immediately onto an ice-cold metal surface to hasten the solidification of the agarose and reduce the time for DNA repair to occur. Once the agarose had set, the slides were carefully submerged in 500 ml of a freshly prepared 50°C lysis solution of 0.5% sodium dodecyl sulphate (SDS) and 30 mM EDTA, pH 8, for 4 h. Subsequently, the slides were rinsed in a solution of TBE buffer (45 mM Tris base, 45 mM boric acid, 2 mM EDTA, pH 8.2) overnight followed by four 15-min washes in fresh TBE buffer. The slides were then transferred to an electrophoresis tank containing 1150 ml of TBE buffer at room temperature. The electrophoresis and washing tanks are made of black perspex, ensuring all light was excluded. Electrophoresis was carried out at room temperature for 25 min at 20 V (~ 0.6 V $cm^{-1}$). Subsequently, the slides were rinsed by submerging in double-distilled water and stained with 2.5 µg $ml^{-1}$ of PI in 0.1 M sodium chloride for 60 min followed by a 30-min rinse in double-distilled water to remove unbound propidium iodide. Slides were dried at room temperature for storage and rehydrated by placing in double-distilled water for 45 min to score. In order to measure residual damage, monolayers of cells in T25 flasks containing 5 ml of culture medium were irradiated at room temperature and immediately returned to a 37°C incubator. After 16 h, the cells were trypsinized, suspended at $8 \times 10^4$ cells $ml^{-1}$ and processed for comet analysis as described above. Experiments measuring the kinetics of DNA dsb rejoining using the most radiosensitive (ME180) and radioresistant cell line (MS751) indicated that small differences were evident in the rate of break rejoining at times < 4 h, however no differences were detected after a 12-h repair period. Consequently, to ensure sufficient time for all the 'rejoinable' breaks to rejoin, a 16-h repair period was used in experiments measuring the absolute proportion of residual DNA dsbs. In addition, 16 h is experimentally appropriate for the development of a routine predictive radiosensitivity test using the neutral comet assay, as it represents an overnight time interval.

### Analysis of initial and residual damage

A minimum of three independent experiments were carried out per cell line. Comets were analysed using a Leitz Diaplan fluorescent microscope at 200× magnification using a Kinetic Imaging Komet system (Liverpool, UK) (Ashby et al, 1995). Tail moment was used as an index of DNA damage, which combines a measure of the length of the comet tail and the proportion of DNA to migrate into the tail (Olive and Banáth, 1993). Before comet scoring, the control sample slide (unirradiated) was examined manually and the brightest comet used to calibrate the CCD camera by ensuring the fluorescence of the comet head did not exceed 255 grey scales. This procedure ensured that subsequent measurements of other comet images did not saturate the CCD camera preventing incorrect head to tail ratios being scored. Comets were measured by randomly selecting images from the microscope field of view and a defined sequence of searching guaranteed that the same image was not scored twice. The mean tail moment value was calculated from 100 comets on two replicate slides per dose point in each of the three individual experiments using the Kinetic Imaging analysis software. To obtain an overall mean tail moment value from the repeat experiments, the mean values from the individual experiments were averaged and standard error on the mean calculated and plotted.

### Flow cytometry

Immediately before each experiment an aliquot of the cell population was washed twice in phosphate-buffered saline (PBS) and resuspended in 0.5 ml of PBS and fixed with 4.5 ml of ice-cold 70% ethanol (in PBS) for a minimum of 24 h at 4°C. Subsequently, the cells were washed twice in PBS, pelleted and resuspended in 800 µl of PBS, 100 µl of RNAase (1 mg $ml^{-1}$) and 100 µl of propidium iodide (1 mg $ml^{-1}$) for 30 min at 37°C (Ormerod, 1990). Analysis of cell cycle phase distributions were carried out on a Becton Dickinson FACScan flow cytometer at 488 nm using a long-pass filter.

### RESULTS

The radiation survival curve parameters after high- (HDR) and low-dose-rate (LDR) irradiation for the seven cell lines used are given in Table 1, and the data are illustrated in Figure 1. The data

© Cancer Research Campaign 1998

**Table 1** Parameters from the linear quadratic model fitted to the survival data in Figure 1 and the mathematically derived $SF_2$ values

| | High-dose-rate survival parameters | | | Low-dose-rate survival parameters | | |
|---|---|---|---|---|---|---|
| **Cell line** | **$SF_2$ ± (95% CL)** | **α ± (95% CL)** | **β ± (95% CL)** | **$SF_2$ ± (95% CL)** | **α ± (95% CL)** | **β ± (95% CL)** |
| MS751 | 0.78 ± 0.09 | 0.07 ± 0.03 | 0.03 ± 0.01 | 0.87 ± 0.18 | 0.06 ± 0.05 | 0.01 ± 0.01 |
| ME180 | 0.31 ± 0.06 | 0.56 ± 0.05 | 0.01 ± 0.02 | 0.38 ± 0.07 | 0.42 ± 0.01 | 0.03 ± 0.01 |
| C33A | 0.55 ± 0.16 | 0.25 ± 0.05 | 0.03 ± 0.01 | ND | ND | ND |
| C41 | 0.61 ± 0.02 | 0.15 ± 0.03 | 0.05 ± 0.01 | 0.74 ± 0.03 | 0.12 ± 0.08 | 0.04 ± 0.02 |
| HT3 | 0.38 ± 0.10 | 0.41 ± 0.04 | 0.03 ± 0.01 | 0.72 ± 0.23 | 0.12 ± 0.03 | 0.03 ± 0.01 |
| SiHa | 0.75 ± 0.05 | 0.13 ± 0.12 | 0.02 ± 0.05 | 0.60 ± 0.14 | 0.25 ± 0.11 | 0.01 ± 0.02 |
| CaSki | 0.87 ± 0.06 | 0.09 ± 0.06 | 0.08 ± 0.02 | 0.73 ± 0.08 | 0.08 ± 0.02 | 0.04 ± 0.01 |

**Figure 1** Radiation survival curves for the cervical tumour cell lines after HDR (right panel) and LDR (left panel) irradiation. The lines are a linear quadratic fit to the data points for HDR irradiation represent the mean survival (± standard deviation) calculated from a minimum of three independent experiments ($n$ = 3–12) with three to six replicate dose measurements; survival after LDR irradiation was calculated from two or three independent experiments. A range of cellular radiosensitivity was seen, with the $SF_2$ values differing by a factor of 2.8 (range 0.31–0.87) after HDR irradiation and by a factor of 2.3 (range 0.38–0.87) after LDR radiation treatment.

Figure 2 shows the initial and residual DNA dsb dose–response curves obtained from the three individual experiments for two of the tumour cell lines used. Linear dose–response curves were evident in each experiment for initial and residual damage. A linear relationship between dose and initial and residual DNA dsbs was also seen in the majority of the individual experiments for the other five cell lines, albeit with greater variability (correlation coefficients ≥0.74) (data not shown). In all experiments, the influence of cell age on assay variability was minimized by using confluent cultures to reduce the proportion of cells in S-phase, as the presence of replication forks in DNA can restrict migration under electrophoresis (Olive et al, 1992; 1993). In each experiment, flow cytometry analysis indicated that the proportion of cells in the S-phase of the cell cycle was always below 18% (range 7–17%, mean 12%), irrespective of the cell line used.

The combined initial and residual DNA dsb dose–response curves compiled from the data of the individual experiments for all seven cell lines are shown in Figure 3. For each cell line, the data points represent the mean (± s.e.m.) tail moment (TM) value calculated from the average TM values from the individual experiments

© Cancer Research Campaign 1998

**Figure 2** Dose–response curves for initial (- - -) and residual (—) DNA dsbs measured using the neutral comet assay in the three individual experiments for the HT3 (upper panels) and SiHa (lower panels) cervical tumour cell lines. Each point represents the mean (± s.d.) tail moment value calculated from two replicate slides per dose point per individual experiment

**Figure 3** Dose–response curves for initial (- - -) and residual (—) DNA dsbs measured using the neutral comet assay. Each point represents the mean (± s.e.m.) calculated from replicate independent experiments. The lines are fitted by linear regression and are fitted by linear regression. The combined data shown in Figure 3 were all well described by a linear fit, with correlation coefficients of 0.96 or higher.

The slopes of the fitted linear regression to the initial DNA dsb dose–response data varied by a factor of 2.9 (range 0.23–0.66) and were steeper than those for residual damage. The shallower residual

© Cancer Research Campaign 1998

**Table 2** Slopes (± 95% CL) from linear regression analysis of the initial and residual dose–response curves in Figure 3. Ratio of damage is calculated as residual damage/initial damage

| Cell line | Initial damage | Residual damage | Ratio |
|---|---|---|---|
| MS751 | 0.66 ± 0.04 | 0.31 ± 0.04 | 0.47 |
| ME180 | 0.58 ± 0.02 | 0.58 ± 0.05 | 0.99 |
| C33A | 0.23 ± 0.02 | 0.16 ± 0.03 | 0.72 |
| C41 | 0.32 ± 0.02 | 0.22 ± 0.01 | 0.68 |
| HT3 | 0.30 ± 0.01 | 0.18 ± 0.01 | 0.61 |
| SiHa | 0.36 ± 0.02 | 0.21 ± 0.02 | 0.58 |
| CaSki | 0.29 ± 0.03 | 0.87 ± 0.01 | 0.53 |

damage slopes indicate that there were fewer DNA dsbs after a 16-h incubation at 37°C (Table 2). The slopes of the fitted linear regression to the residual DNA dsb dose–response curves varied by a factor of 3.6 (range 0.16–0.58). The ratio of the slopes of the initial and the residual dose–response curves, representing the fraction of DNA damage unrepaired, varied by a factor of 2.1 (range 0.47–0.99).

A comparison was made of the relationship between clonogenic survival after HDR and LDR irradiation and the level of radiation-induced DNA dsbs measured using the neutral comet assay. No relationship was found between HDR $SF_2$ and the slopes of initial ($P$ = 0.91) or residual ($P$ = 0.23) DNA dsb dose–response curves fitted by linear regression. In contrast, an association was evident between HDR $SF_2$ and the ratio of the number of initial and residual DNA dsbs (i.e. the fraction of DNA damage unrepaired) after a 60-Gy dose of radiation ($r$ = 0.58; $P$ = 0.17) (Figure 4B). Moreover, a statistically significant correlation was found between HDR $SF_2$ and the ratio of initial and residual DNA dsbs over the complete dose range examined, as represented by the slope parameters calculated from Figure 3 ($r$ = –0.78; $P$ = 0.04) (Figure 4A). Stronger correlations were found between $SF_2$ after low-dose-rate irradiation and the fraction of DNA damage unrepaired (ratio of initial and residual damage) at 60 Gy ($r$ = 0.78; $P$ = 0.09) and over the complete dose range studied ($r$ = –0.86; $P$ = 0.03) (Figure 4C and D).

### DISCUSSION

Evidence that the radiosensitivity of a tumour is an important factor involved in determining patient response to radiotherapy has been gained from work carried out using clonogenic assays (West, 1995; West et al, 1997). These assays are however too laborious for routine clinical application, and this has stimulated an interest in the development of more rapid measures of radiosensitivity. The comet assay is attractive as a potential clinical test of tumour radiosensitivity as it requires few cells and generates results rapidly compared with standard clonogenic and other electrophoresis assays.

The linear relationship between the production of radiation-induced DNA dsbs and dose shown in Figures 2 and 3 is in good agreement with previous studies using a variety of DNA dsb assays (reviewed by Núñez, 1996) and the neutral comet assay (Olive et al, 1994). In the present study, a comparison of the slopes obtained from the linear regression analysis of the initial DNA dsb dose–response curves for the seven tumour lines showed significant variation, indicating different levels of initial damage between

**Figure 4** Relationship between ratio of damage (initial/residual) at 60 Gy (**B** and **D**) or ratio of slopes of the initial and residual DNA dsb dose–response curves and radiosensitivity expressed as $SF_2$ for HDR (**A** and **B**) and LDR (**C** and **D**) irradiation for the series of cervical tumour cell lines. The solid lines represent the regression fit to the data and the dashed lines the 95% confidence on the fit

© Cancer Research Campaign 1998

**Table 3** Correlation coefficients and *P*-values calculated from either HDR or LDR $SF_2$ plotted against the slopes of the initial and residual damage dose–response curves, the ratio of slopes of residual and initial damage dose–response curves or the ratio of the absolute level of initial residual level of DNA dsbs at 60 Gy (as determined from Figure 3)

| | Initial slope | | Residual slope | | Ratio of slopes | | Ratio at 60 Gy | |
|---|---|---|---|---|---|---|---|---|
| | *r* | *P* | *r* | *P* | *r* | *P* | *r* | *P* |
| HDR $SF_2$ | –0.05 | 0.91 | –0.51 | 0.23 | –0.78 | 0.04* | 0.58 | 0.17 |
| LDR $SF_2$ | –0.07 | 0.89 | –0.59 | 0.21 | –0.86 | 0.03* | 0.78 | 0.09** |

*Correlation is significant at the level 0.05. **Correlation is significant at the level 0.10.

the different cell lines (Figure 3 and Table 2). The present finding agrees with other studies that have shown significant differences in DNA dsb induction between human tumour cells lines using neutral elution (Kelland et al, 1988) or pulsed-field gel electrophoresis (Whitaker et al, 1995). However, the results from Figure 3 contrast with the data of Olive et al (1994) using the neutral comet assay which showed no significant differences in radiation-induced DNA dsb in a range of six histologically distinct human tumour cell lines (melanoma, prostate carcinoma, glioma, colon adenocarcinoma and cervical carcinoma).

In order to have confidence in the potential use of a rapid assay of tumour radiosensitivity, correlations must be shown with clonogenic measures of radiosensitivity. A significant correlation has been reported previously between clonogenic radiosensitivity and the levels of initial radiation-induced damage in human tumour cells (Kelland et al, 1988; Núñez et al, 1996). It was postulated that this might reflect differences in the number of lesions incurred by the radiosensitive cells compared with the resistant cells, as shown by Peacock et al (1989), or possibly differences in chromatin conformation that affect the accessibility of scavenging molecules to damaged DNA or radical attack (Oleinick et al, 1984; Olive et al, 1992) or the 'presentation' of DNA damage sites (Woudstra et al, 1996). However, the results from this study indicate that in the cervical tumour lines studied here the level of initial DNA damage does not correspond with clonogenic radiosensitivity after HDR and LDR irradiation (Table 3).

In contrast, Ward (1990) argued that the yield of molecular lesions (initial damage) is independent of tumour cell type and that radiation-sensitive cells are repair deficient, resulting in different levels of residual damage. However, in our work, there were no significant differences in the slopes of the residual DNA dsb dose–response curves fitted by linear regression and $SF_2$ after HDR and LDR irradiation. Nevertheless other human tumour studies measuring DNA dsbs by asymmetric field-inversion gel electrophoresis (Giaccia et al, 1992) and neutral filter elution (Zaffaroni et al, 1994) have indeed shown significant correlations between the level of residual dsbs and radiosensitivity.

A new finding in the present studies was the significant correlation with the extent of dsb repair (ratio of initial and residual slopes) after 16 h of post-irradiation incubation and radiosensitivity after HDR ($r = -0.78$, $P = 0.04$) or LDR ($r = -0.86$, $P = 0.03$) irradiation (Figure 4). DNA dsb repair measured using the neutral comet assay and cellular radiosensitivity were found not to correlate in a study by Olive et al (1992), however one possible explanation for the difference between the present study and that of Olive et al (1992) may reflect the time interval allowed for repair: 4 h in the previous study and 16 h in the present study. It has been noted that measuring residual DNA dsbs using the neutral comet assay at 4 h may underestimate the level of DNA damage as a result of the increase in the percentage of S-phase cells after irradiation, as S-phase DNA migrates inefficiently. This would not be expected in the present study using a 16 h period of repair (Olive et al, 1994).

In support of the correlation between $SF_2$ and repair capacity reported here, a non-significant trend was evident between LDR $SF_2$ and the absolute difference in residual and initial DNA dsbs assessed from a single dose of 60 Gy ($P = 0.09$) and a significant trend over the complete dose range studied ($P = 0.03$) (Figure 4B and D). The stronger relationship between the level of DNA dsbs and $SF_2$ measured after LDR irradiation is not unexpected and reflects the potential of each cell line to repair radiation-induced DNA dsbs during exposure. A correlation between radiosensitivity and level of dsbs in four histologically related tumour cell lines has been reported previously after low-dose-rate but not at high-dose-rate irradiation using PGFE (Cassoni et al, 1992).

The approach of examining the unrepaired DNA dsb fraction by taking a ratio of dose–response curve slopes (thereby assessing damage from a range of multiple dose points) minimizes variability between experiments on the different cell lines and suggests that it is only by doing so that a significant correlation can be seen between HDR $SF_2$ and DNA dsb repair capacity as measured by the neutral comet assay. Recently, we have also found a strong correlation between the ratio of slopes of initial/residual DNA dsb dose–response curves and radiosensitivity after HDR irradiation for nine normal fibroblast strains derived from vaginal biopsies from pretreatment cancer patients ($r = 0.80$, $P = 0.01$) (Marples et al, 1997). It is not clear yet whether the importance of the unrepaired fraction of DNA dsbs is related to, for example, DNA ploidy, possible influence of chromatin structure/DNA organization on the translation of residual DNA damage into potentially lethal events or on the tolerance of cells to genetic injury. Alternatively, assessing repair capacity by the ratio of damage (initial/residual) may relate to misrepair of radiation-induced DNA dsbs. Such possibilities will require further investigation.

A review of the literature (see Introduction) illustrates the disparate findings over the possible relationship between DNA dsb damage and clonogenic measures of radiosensitivity. There is clearly some relationship but the question of interest is which parameter is the most important component (initial or residual damage, ratio of damage, rate of repair, time at which repair is assayed, etc.). The balance of evidence suggests that residual damage is likely to be the most critical aspect but, in view of the published data, the significance of initial damage cannot yet be discounted. The divergent results may relate to differences in the assays used to score DNA dsb damage and/or experimental design (e.g. selection and number of cell lines, doses used, time allowed for repair). In an attempt to overcome some of the limitations of other studies, we have selected a large series of cell lines of the same histological origin (seven human cervical carcinoma cell lines) and have assessed both initial and residual DNA damage over a range of radiation doses. In doing so, we have demonstrated a significant relationship between the ratio of initial and residual radiation-induced DNA dsbs, measured using the neutral comet assay, and cellular radiosensitivity measured using a clonogenic assay. It is likely that the continued improvements of assay conditions may improve this further, e.g. more sensitive DNA-binding dyes, more optimal lysing conditions. The work supports the

© Cancer Research Campaign 1998

continued use of the neutral comet assay as a potential rapid predictive test for tumour radiosensitivity in radiotherapy.

### ACKNOWLEDGEMENTS

This work was supported by the Association for International Cancer Research and the Cancer Research Campaign, UK. The assistance of Mr J Barry in carrying out the flow cytometric measurements is acknowledged. We particularly thank Dr Peggy Olive (Medical Biophysics, British Colombia Cancer Research Centre, Vancouver, Canada) for discussion of the comet assay data. Also, the authors would like to thank the staff and customers at Asda (Chadderton, UK) for funds to purchase the image analysis system.

### REFERENCES

Ashby J, Tinwell H, Lefevre PA and Browne MA (1995) The single cell gel electrophoresis assay for induced DNA damage (comet assay): measurement of tail length and moment. *Mutagenesis* **10**: 85–90

Cassoni AM, McMillan TJ, Peacock JH and Steel GG (1992) Differences in the level of DNA double-strand breaks in human tumour cell lines following low dose-rate irradiation. *Eur J Cancer* **28A**: 1610–1614

Fairbairn DW, Olive PL and O'Neill KL (1995) The comet assay: a comprehensive review. *Mutat Res* **339**: 37–59

Giaccia AJ, Schwartz J, Shieh J and Brown JM (1992) The use of asymmetric-field inversion gel electrophoresis to predict tumor cell radiosensitivity. *Radiother Oncol* **24**: 231–238

Kelland LR, Edwards SM and Steel GG (1988) Induction and rejoining of DNA double-strand breaks in human cervix carcinoma cell lines of differing radiosensitivity. *Radiat Res* **116**: 526–538

Kiltie AE, Orton CJ, Ryan A, Roberts SA, Marples B, Davidson SE, Hunter RD, Margison G, West CML and Hendry JH (1997) A correlation between DNA damage and clonogenic measurements of radiosensitivity in fibroblasts from pre-radiotherapy cervix cancer patients. *Int J Radiat Oncol Biol Phys* **39**: 1137–1144

McKay ML and Kefford RF (1995) The spectrum on *in vitro* radiosensitivity in four human melanoma cell lines is not accounted for by differential induction or rejoining of DNA double-strand breaks. *Int J Radiat Oncol Biol Phys* **31**: 345-352

McMillan TJ, Cassoni AM, Edwards S, Holmes A and Peacock JH (1990) The relationship of DNA double-strand break induction to radiosensitivity in human tumour cell lines. *Int J Radiat Biol* **58**: 427–438

Marples B and Joiner MC (1993) The response of Chinese hamster V79 cells to low radiation doses: evidence of enhanced sensitivity of the whole cell population. *Radiat Res* **133**: 41–51

Marples B, Eastham AM, Kiltie AE, Orton JC and West CML (1997) Fibroblast radiosensitivity measured using the comet assay correlates with clonogenic survival parameters. (In preparation)

Nunez MI, McMillan TJ, Valenzuela MT, Ruiz de Almodovar JM and Pedraza V (1996) Relationship between DNA damage, rejoining and cell killing by radiation in mammalian cells. *Radiother Oncol* **39**: 155–165

Oleinick NL, Chiu S and Friedman LR (1984) Gamma irradiation as a probe of chromatin structure: damage and repair of chromatin in the metaphase chromosome. *Radiat Res* **98**: 629–641

Olive PL and Banath JP (1993) Induction and rejoining of radiation induced DNA single-strand breaks: tail moment as a function of position in the cell cycle. *Mutat Res (DNA repair)* **294**: 275–283

Olive PL, Wlodek D and Banath JP (1991) DNA double strand breaks measured in individual cells subjected to gel electrophoresis. *Cancer Res* **51**: 4671–4676

Olive PL, Durand RE, Wlodek D and Banath JP (1992) Factors influencing DNA migration from individual cells subjected to gel electrophoresis. *Exp Cell Res* **198**: 259–267

Olive PL, Banath JP and MacPhail HS (1994) Lack of a correlation between radiosensitivity and DNA double-strand break induction or rejoining in six human tumour cell lines. *Cancer Res* **54**: 3939–3946

Ormerod MG (1990) Analysis of DNA: general methods. In *Flow Cytometry: A Practical Approach*, Ormerod MG. (ed.), pp. 69–87. Oxford University Press: New York

Peacock JM, Eady JJ, Edwards S, Holmes A, McMillan TJ and Steel GG (1989) Initial damage and repair as the major determinant of cellular radiosensitivity. *Int J Radiat Biol* **56**: 543–547

Powell SN and McMillan TJ (1994) The repair fidelity of restriction enzyme-induced double strand breaks in plasmid DNA correlate with radioresistance in human tumour cell lines. *Int J Radiat Oncol Biol Phys* **29**: 1035–1040

Powell SN, Whitaker SJ, Edwards SM and McMillan TJ (1992) A DNA repair defect in a radiation-sensitive clone of a human bladder carcinoma cell line. *Br J Cancer* **65**: 798–802

Ruiz de Almodóvar JMR, Núnez MI, McMillan TJ, Olea N, Mort C, Villalobos M, Pedraza V and Steel GG (1994) Initial radiation-induced DNA damage in human tumour cell lines: a correlation with intrinsic cellular radiosensitivity. *Br J Cancer* **69**: 457–462

Schwartz JL, Rotmensch J, Giovanazzi SM, Cohen MB and Weichselbaum RR (1988) Faster repair of DNA double-strand breaks in radioresistant human tumour cells. *Int J Radiat Oncol Biol Phys* **15**: 907–912

Smeets MF, Mooren EH and Begg AC (1993) Radiation-induced DNA damage and repair in radiosensitive and radioresistant human tumour cells measured by field inversion gel electrophoresis. *Int J Radiat Biol* **63**: 703–713

Ward JF (1990) The yield of DNA double-strand breaks produced intracellularly by ionising radiation: a review. *Int J Radiat Biol* **57**: 1141–1150

West CML (1995) Intrinsic radiosensitivity as a predictor of patient response to radiotherapy. *Br J Radiol* **68**: 827–837

West CML, Davidson SE, Roberts SA and Hunter RD (1997) The independence of intrinsic radiosensitivity as a prognostic factor for patient response to radiotherapy of carcinoma of the cervix. *Br J Cancer* **76**: 1184–1190

Whitaker SJ, Ung Y and McMillan TJ (1995) DNA double-strand break induction and rejoining as determinants of human tumour radiosensitivity. *Int J Radiat Biol* **67**: 7–18

Wilks DP, Barry J, Hughes MF and West CML (1996) Assessment of light scatter by nucleoids as a rapid predictive assay of radiosensitivity. *Radiat Res* **146**: 628–635

Woudstra EC, Brunsting JF, Roesink JM, Konings SW and Kampinga HH (1996) Radiation induced DNA damage repair in three human tumour cell lines. *Mutat Res* **362**: 51–59

Würm R, Burnet NG, Duggal N, Yarnold JR and Peacock JH (1994) Cellular radiosensitivity and DNA damage in primary human fibroblasts. *Int J Radiat Oncol Biol Phys* **30**: 625–633

Zaffaroni N, Orlandi L, Villa R, Bearzatto A, Rofstad EK and Silvestrini R (1994) DNA double-strand break repair and radiation response in human tumour primary cultures. *Int J Radiat Biol* **66**: 279–285

© Cancer Research Campaign 1998